# Teleassessment in Clinical Psychology: Opportunities, Limitations, and Future Developments

**DOI:** 10.1192/j.eurpsy.2026.11355

**Published:** 2026-07-31

**Authors:** G. M. Festa

**Affiliations:** 1 Pontifical Faculty of Educational Sciences «AUXILIUM»; 2 Interdisciplinary Institute of Advanced Clinical Training «IACT», Rome, Italy

## Abstract

**Introduction:**

In recent years, teleassessment has taken on an increasingly important role in psychological practice, emerging as an innovative methodology that employs digital technologies and remote communication tools (videoconferencing, online platforms, dedicated software, etc.) for clinical evaluation, administration of psychodiagnostic tests, and feedback delivery. This approach has contributed to overcoming geographical and logistical barriers and, in many cases, has improved access to psychological services. For further discussion on the topic, see: Festa, G. M. (2025). *Teleassessment in Psychological Practice: Opportunities, Challenges and Critical Considerations*, *Journal of Behavioral Health and Psychology*, 14(4).

**Objectives:**

This paper aims to provide a brief overview of teleassessment by highlighting its main benefits, existing limitations, and ethical implications. It also presents operational guidelines for the correct and responsible use of this methodology, emphasizing the importance of practitioner training and empirical validation of the tools employed.

**Methods:**

A review of international scientific literature on teleassessment in clinical settings was conducted. The review included theoretical articles, empirical studies, and professional guidelines published by psychological associations, with the goal of identifying strengths, limitations, and best practices. Special attention was given to technological, clinical, methodological and ethical aspects.

**Results:**

Teleassessment is proving to be useful in expanding access to psychological services, especially in remote areas or emergency situations (e.g., the COVID-19 pandemic). Key advantages include organizational flexibility, reduced wait times, and lower travel-related costs for clients. However, some limitations persist: the unsuitability of certain instruments for remote administration, and risks concerning data privacy and cybersecurity.

**Conclusions:**

Teleassessment represents a concrete opportunity for innovation in clinical psychological practice, yet it requires specific training for professionals, shared operational guidelines, and validated tools for remote use. In the near future, the evolution of immersive technologies (virtual and augmented reality) may further revolutionize psychological assessment, opening new avenues for intervention and personalized care. A collaborative effort between researchers and clinicians is strongly recommended to ensure accessibility, efficacy and safety.

**Disclosure of Interest:**

None Declared

